# Sociodemographic Disparities and Hearing-Related Quality of Life in Children With Hearing Loss

**DOI:** 10.1001/jamanetworkopen.2023.40934

**Published:** 2023-10-30

**Authors:** Brooke R. Warren, Inderpreet Kaur Khalsa, Jihyun Stephans, Dylan K. Chan

**Affiliations:** 1 Department of Otolaryngology-Head & Neck Surgery, University of California, San Francisco

## Abstract

This cohort study assesses the association of sociodemographic differences with quality of life in deaf and hard-of-hearing children and adolescents in the US.

## Introduction

Hearing loss (HL) is the most common congenital sensory disorder, with 1 in 500 children born deaf or hard-of-hearing (DHH) in the US every year.^1,2^ The Hearing Environments and Reflection on Quality of Life (HEAR-QL) survey is a validated tool that quantifies how a child perceives the social and emotional impact of their HL in multiple areas of daily life.^3,4^ To our knowledge, there are no studies that assess differences in hearing quality of life (QOL) across diverse DHH populations. Therefore, we sought to assess the association of sociodemographic differences with QOL in DHH children.

## Methods

We conducted a retrospective cohort study of patients with permanent HL seen at our tertiary care center from 2014 to 2022. Patients aged 7 to 12 years received the 26-question HEAR-QL Measurement for Children survey, and patients aged 13 to 18 years received the 28-question HEAR-QL Measurement for Adolescents survey. Higher HEAR-QL scores equate to higher hearing-related QOL. Total and subdomain scores were assessed as outcomes. We performed descriptive statistics, as well as linear and multivariable regression. The Strengthening the Reporting of Observational Studies in Epidemiology (STROBE) reporting guidelines were followed. The eAppendix in Supplement 1 details our analytical strategy. The institutional review board (IRB) of the University of California, San Francisco, approved the study and granted a waiver of signed informed consent because of implied consent upon completion of the completed survey. The IRB approved a model whereby families were provided an information sheet about the study and implied consent was determined by virtue of parents completing the survey.

## Results

A total of 583 HEAR-QL surveys were completed by pediatric patients with HL. Of those, 299 patients met inclusion criteria and had complete data for analysis. Sociodemographic factors in Table 1 demonstrate the considerable demographic diversity reflective of the population of the Northern California region and our clinic^5^; 237 patients (79%) were of a race other than White, 124 (42%) identified as Hispanic, 110 (37%) had parents whose primary language was not English, and 206 (69%) were publicly insured. The mean (SD) overall area deprivation index (ADI) ranking was 3.92 (3.1), signifying that 60.8% of Census block groups in California are less socioeconomically disadvantaged than our study population.

**Table 1.** Characteristics of the Study Population With Univariable and Multivariable Linear Regression of Composite Hearing Environments and Reflection on Quality of Life Scores With Sociodemographic and Clinical Variables

Characteristic	Patients, No. (%)			Univariable linear regression		Multivariable linear regression^a^	
	All (N = 299)	Aged 7-12 y (n = 196)	Aged 13-18 y (n = 103)	Coefficient (95% CI)	*P* value^b^	Coefficient (95% CI)	*P* value
Age, mean (SD), y	12.0 (3.1)	10.2 (2.0)	15.3 (1.8)	−0.6 (−1.4 to 0.1)	.10	−0.4 (−1.2 to 0.4)	.34
Gender							
Male	154 (52)	103 (53)	51 (50)	2.8 (−1.8 to 7.4)	.23	4.9 (−0.2 to 10.0)	.60
Female	145 (48)	93 (47)	52 (50)				
Primary language							
English	189 (63)	127 (65)	62 (61)	2.2 (−2.6 to 7.0)	.36	0.6 (−5.7 to 6.8)	.85
Any language other than English	110 (37)	69 (35)	41 (39)				
Insurance type							
Private	93 (31)	67 (34)	26 (26)	−1.7 (−6.7 to 3.3)	.50	−0.9 (−7.5 to 5.6)	.78
Public	206 (69)	129 (66)	77 (74)				
Race and ethnicity^c^							
Hispanic	124 (42)	80 (41)	44 (42)	−1.6 (6.3 to 3.0)	.48	−1.8 (−7.5 to 3.9)	.53
Non-Hispanic							
American Indian or Alaska Native	3 (1)	3 (2)	0	−11.0 (−34.0 to 12.0)	.35	−17.4 (−54.5 to 19.8)	.36
Asian	53 (18)	37 (19)	16 (15)	9.9 (4.1 to 15.8)	.001	7.1 (0.14 to 14.1)	.046
Black	12 (4)	5 (3)	7 (7)	3.4 (−8.3 to 15.1)	.57	1.0 (−10.2 to 12.3)	.86
Native Hawaiian or Other Pacific Islander	5 (2)	1 (<1)	4 (4)	3.6 (−14.3 to 21.4)	.70	6.3 (−15.3 to 28.0)	.57
White	62 (21)	38 (19)	24 (23)	−9.2 (−14.8 to −3.6)	.001	−8.3 (−15.5 to −1.0)	.03
Other^d^	43 (14)	32 (16)	11 (1)	0.1 (−6.4 to 6.6)	.98	−0.3 (−7.4 to 6.7)	.93
Laterality							
Unilateral	67 (22)	43 (22)	24 (23)	−3.9 (−9.5 to 1.8)	.18	4.8 (−2.0 to 11.6)	.16
Bilateral	178 (60)	122 (62)	56 (54)				
Area deprivation index, mean (SD), percentile	3.92 (3.1)	3.89 (3.1)	3.96 (3.0)	NA	NA	NA	NA
Comorbidities, mean (SD), No.	2.65 (2.20)	2.68 (2.10)	2.58 (2.40)	−0.6 (−1.7 to 0.5)	.26	0.3 (−0.9 to 1.5)	.62
Pure tone average, mean (SD), dB hearing loss^e^	30.2 (25.6)	30.0 (24.2)	30.6 (21.8)	−0.2 (−0.3 to −0.1)	<.001	−0.2 (−0.4 to −0.1)	.001

Abbreviation: NA, not applicable.

^a^ Covariates include gender, insurance type, primary home language, race and ethnicity (dichotomized as non-Hispanic White vs all other races), laterality, number of comorbidities, and pure tone average.

^b^ Bonferroni correction was applied for exploratory univariable analysis, and adjusted statistical significance threshold was set at *P* < .005.

^c^ For each racial and ethnic category, we compared the indicated racial and ethnic group with all other patients not in that racial and ethnic group.

^d^ Other refers to multiracial or any other race or ethnicity not listed.

^e^ Refers to unaided pure tone average of the better-hearing ear.

For univariable analyses (Table 1), gender, insurance type, and primary home language were not associated with HEAR-QL score. To account for differences in HL, we also evaluated the association of laterality (unilateral vs bilateral HL) and hearing level (pure tone average [PTA] in the better-hearing ear) with HEAR-QL scores. Although laterality was not significant, PTA had an inverse association with HEAR-QL score; lower PTA (ie, less HL) was associated with higher HEAR-QL score. Non-Hispanic White race and ethnicity was associated with lower HEAR-QL score on both univariable and multivariable regression accounting for all sociodemographic and clinical covariates. In contrast, non-Hispanic Asian race and ethnicity was associated with higher HEAR-QL scores on both univariable and multivariable regression. PTA of the better hearing ear remained significantly associated with HEAR-QL composite score on multivariable regression.

ADI ranking was negatively associated with QOL on multivariable analysis (Table 2). For the composite HEAR-QL score, patients with a high ADI ranking, representing greater socioeconomic disadvantage, were significantly more likely to have a lower HEAR-QL score. For patients aged 7 to 12 years, although the total HEAR-QL score was not significantly associated with ADI, ADI was negatively associated with the Environment subsection QOL score. For patients aged 13 to 18 years, high ADI was significantly associated with low total HEAR-QL score as well as all 3 of 4 subsections: Feelings, Social Interaction, and School Difficulties.

**Table 2.** Multivariable Linear Regression of HEAR-QL Scores With Area Deprivation Index Scores With Sociodemographic and Clinical Variables^a^

HEAR-QL category	Coefficient (95% CI)	*P* value^b^
Composite	−1.5 (−2.3 to 0.9)	<.001
Measurement for Children survey (patients aged 7-12 y)		
Total	−0.9 (−1.9 to 0.2)	.09
Feelings	−0.9 (−2.3 to 0.6)	.25
Environment	−1.7 (−2.9 to −0.5)	.006
Activities	0.4 (−0.9 to 1.7)	.58
Measurement for Adolescents survey (patients aged 13-18 y)		
Total	−2.6 (−4.3 to −0.9)	.004
Feelings	−3.0 (−5.2 to −1.0)	.005
Social Interactions	−2.6 (−4.3 to −0.9)	.003
Hearing Situations	−1.6 (−3.7 to 0.4)	.11
School Difficulties	−3.1 (−5.2 to −1.0)	.004

Abbreviation: HEAR-QL, Hearing Environments and Reflection on Quality of Life.

^a^ Covariates include gender, insurance type, primary home language, race and ethnicity (dichotomized as non-Hispanic White vs all other races and ethnicities), laterality, number of comorbidities, and pure tone average.

^b^ Statistical significance threshold was set at *P* < .05.

## Discussion

The findings of this cohort study suggest that race and ethnicity and neighborhood disadvantage are associated with hearing-related QOL in DHH children. The neighborhood association was seen most broadly in children older than 13 years. Limitations include response bias and inability to know the underlying modifiable factors that neighborhood vulnerability and what non-Hispanic White and Asian race and ethnicity are proxies for in their associations with QOL. Future studies should elucidate these modifiable factors to enable multidisciplinary health care teams to provide more tailored HL care and improve QOL in DHH children.

## Notes

Supplement 1. **eAppendix.** Supplemental Methods
eReference

Supplement 2.
Data Sharing Statement
