# Supplementary material for: Sociodemographic Disparities and Hearing-Related Quality of Life in Children With Hearing Loss
Source: JAMA Netw Open. 2023 Oct 30;6(10):e2340934. doi: 10.1001/jamanetworkopen.2023.40934 (PMC10616717; doi:10.1001/jamanetworkopen.2023.40934)
Supplement: Supplement 1. — eAppendix. Supplemental Methods eReference [file jamanetwopen-e2340934-s001.pdf]

Supplemental Online Content

Warren BR, Kaur Khalsa I, Stephans J, Chan DK. Sociodemographic disparities and hearing-related quality of life in children with hearing loss. *JAMA Netw Open*. 2023;6(10):e2340934. doi:10.1001/jamanetworkopen.2023.40934

eAppendix. Supplemental Methods
eReference

This supplemental material has been provided by the authors to give readers additional information about their work.

© 2023 Warren BR et al. *JAMA Network Open.*

eAppendix. Supplemental Methods

We conducted a cohort study on patients who have ever received care for permanent HL in the multidisciplinary Hearing and Communication Clinic (comprising pediatric otolaryngology, audiology, speech-language pathology, and social work specialists) at a tertiary children's hospital in San Francisco, California from 2014 to 2022. All patients seen at this clinic were audiologic candidates for hearing devices and had a diagnosis of permanent hearing loss, either sensorineural, mixed, or permanent conductive. Data analyses were performed from November 2022 to March 2023.

Patients 7 to 12 years of age received the 26-question HEAR-QL Measurement for Children survey and patients 13 to 18 years of age received the 28-question HEAR-QL for Measurement for Adolescents survey. The surveys were administered before or after a visit to the multidisciplinary Hearing and Communication Clinic using either a paper form or electronic version on REDcap. The HEAR-QL is only available in English. If a patient or their parent's preferred language was non-English, an in-person or virtual certified medical interpreter was provided for translation of the survey questions. Additionally, for patients who were 7 to 12 years of age, parents were asked to complete the surveys with their child while children aged 13 to 18 were asked to complete on their own if they were able.

The 26-question HEAR-QL Measurement for Children survey is composed of 3 sections: Environments (13 questions), Activities (6 questions), and Feelings (7 questions). The 28-question HEAR-QL for Measurement for Adolescents survey is

© 2023 Warren BR et al. *JAMA Network Open.*

composed of 4 sections: Hearing Situations (6 questions), Social Interactions (7 questions), School Difficulties (7 questions), and Feelings (8 questions). Each question response was coded using a Likert scale including “never” (1), “almost never” (2), “sometimes” (3), “often” (4), and “almost always” (5). These scores were subsequently converted to a 100-point scale where 1=100, 2=75, 3=50, 4=25, and 5=0. The scores for each question within a subsection were then averaged to give the subsection score. To get the composite score, the subsection scores were averaged together. Therefore, for the Measurement for Children HEAR-QL survey, 3 subsection scores were averaged together to form the composite score and for the Measurement for Adolescents HEAR-QL survey, 4 subsection scores were averaged together to form the composite score.

Patients were excluded if they did not complete a complete sociodemographic survey. If a patient had completed more than one HEAR-QL survey, the first survey completed was used. Additionally, if a patient had completed both the survey for 7-to-12-year-olds and 13-to-18-year-olds, we included only the first survey (7-to-12-year-old survey).

Data were extracted from the electronic medical record. Gender, ethnicity and race, insurance type, and primary language of medical communication of the parents were based on parental report. Number of comorbid conditions was determined as the number of ICD-10 codes on the child’s problem list. Hearing-loss laterality and air-conduction pure-tone average of the better hearing ear was extracted from the earliest available audiogram. Area deprivation index (ADI) percentile was determined from residential street address. ADI is a validated placed-based measure of socioeconomic

disadvantage, which weighs 17 US Census-derived social determinants of health, including education, employment, housing quality, and poverty variables, and provides an overall decile ranking (ranging from 0-10, with 10 being the most deprived). To determine ADI, residential addresses were obtained from the electronic medical record and geocoded to a census block group using 2020 US Census boundaries; census block groups are considered the closest approximation of a “neighborhood” and are the smallest geographic unit (typically containing 600-3,000 residents) with data reported by the Census bureau. After census block group identification, each participant was assigned corresponding 2020 California state ADI decile rankings.[1] Race and ethnicity were categorized together, as follows. All patients who indicated that they were Hispanic or Latinx were recorded as Hispanic; all patients who indicated that they were non-Hispanic or Latinx and Black were recorded as non-Hispanic Black (NH Black); all patients who identified as Asian and not in the aforementioned categories were recorded as non-Hispanic Asian (NH Asian); all patients who identified as White and not in the aforementioned categories were recorded as White (NH White); all patients who identified as American Indian/Alaska Native were recorded as non-Hispanic American Indian/Alaska Native (NH AI/AN); all patients who identified as Native Hawaiian/Pacific Islander were recorded as non-Hispanic Native Hawaiian/Pacific Islander (NH NHPI); and all the remaining patients were recorded as Other. Hearing level was measured as ear-specific pure-tone average (PTA) of the air-conduction thresholds at 0.5, 1, 2, and 4 kHz. Hearing loss was considered unilateral if the better-ear PTA was < 20 dB HL.

© 2023 Warren BR et al. *JAMA Network Open.*

Descriptive statistics were performed to describe the cohort. Continuous independent and dependent variables were compared with linear and multivariate regression. All statistical tests were completed using Stata, version 18. For initial exploratory univariate analysis, a Bonferroni correction to the statistical significance threshold was made at $p<0.005$ to account for the 9 comparisons (gender, primary language of medical communication of the parents, insurance type, non-White race/ethnicity, number of comorbid conditions, laterality, pure tone average of better hearing ear, and ADI). We also performed multivariate logistic regression to evaluate the association of sociodemographic variables with HEAR-QL scores where the statistical significance threshold was $p<0.05$. The Bonferroni correction was not applied to the multivariate regression, which was performed as a follow-up to the univariate analysis.

eReference

1. University of Wisconsin School of Medicine and Public Health. *Area Deprivation Index 2.0.*; 2020. Accessed March 6, 2023.

© 2023 Warren BR et al. *JAMA Network Open.*
