# Supplementary material for: Sociodemographic Disparities and Hearing-Related Quality of Life in Children With Hearing Loss
Source: JAMA Netw Open. 2023 Oct 30;6(10):e2340934. doi: 10.1001/jamanetworkopen.2023.40934 (PMC10616717; doi:10.1001/jamanetworkopen.2023.40934)
Supplement: Supplement 2. — Data Sharing Statement [file jamanetwopen-e2340934-s002.pdf]

Data Sharing Statement

Warren. Sociodemographic Disparities and Hearing-Related Quality of Life in Children With Hearing Loss. *JAMA Netw Open*. Published October 30, 2023.
doi:10.1001/jamanetworkopen.2023.40934

Data
Data available: Yes
Data types: Deidentified participant data
How to access data: Dylan.chan@ucsf.edu
When available: With publication

Supporting Documents
Document types: None

Additional Information
Who can access the data: Anyone requesting
Types of analyses: For any purpose
Mechanisms of data availability: With investigator support
